# Novel monoclonal antibodies for an activated protein C cleavage neoepitope of coagulation factor Va

**DOI:** 10.1016/j.rpth.2026.106963

**Published:** 2026-09-10

**Authors:** Keiko Maruyama, Koichi Kokame

**Affiliations:** Department of Molecular Pathogenesis, National Cerebral and Cardiovascular Center, Suita, Japan

Coagulation factor (F)Va is the cofactor component of the prothrombinase complex and facilitates the conversion of prothrombin to thrombin, a central reaction in efficient blood clotting [1]. FVa is generated from the inactive procofactor FV by proteolytic removal of the large central B domain. To prevent excessive clot formation, FVa cofactor activity is downregulated by activated protein C (APC) and its cofactor protein S (PS), which cleave FVa at Arg^306^, Arg^506^, and Arg^679^. Cleavage at Arg^306^ is required for complete FVa inactivation [2,3]. The physiological importance of this pathway is underscored by the increased risk of venous thromboembolism associated with PC deficiency, PS deficiency, and the FV Leiden (Arg506Gln) mutation [4,5].

Here, we developed monoclonal antibodies (mAbs) that specifically recognize the newly exposed Asn^307^ neoepitope of FV generated by APC-mediated cleavage at Arg^306^. The mAbs were generated using Ecobody technology protocols by iBody Inc [6]. In brief, a rabbit was immunized with a keyhole limpet hemocyanin-conjugated target peptide (NLKKITRE+C, corresponding to Asn^307^-Glu^314^ of human FV plus Cys), and lymphocytes were collected from peripheral blood. B cells showing high reactivity to the target peptide and low reactivity to the control peptide (KKTRNLKKITRE+C, corresponding to Lys^303^-Glu^314^ of human FV plus Cys) were isolated by fluorescence-activated cell sorting. Antibody light- and heavy-chain variable region genes were amplified by polymerase chain reaction from complementary DNA prepared from each cell, and Fab fragments were expressed using a cell-free protein synthesis system. The reactivity of each expressed Fab fragment to the target and control peptides was tested by enzyme-linked immunosorbent assay (ELISA). Two clones, 58CC07 and 58CC09, reacted specifically with the target peptide. The nucleotide sequences of their variable regions were determined by Sanger sequencing and subcloned into rabbit immunoglobulin G expression vectors. Recombinant mAbs were expressed in human embryonic kidney 293 cells by transient transfection and purified from culture supernatants.

We confirmed that 58CC07 and 58CC09 specifically reacted with APC-cleaved fragments of purified human FVa (hFVa) and modified FV (FVm). FVm is a B-domain–deleted FV mutant that mimics FVa and was constructed by deleting Arg^709^-Arg^1545^ and inserting a His tag and linkers in place of this region; we previously used FVm in a plasma PS activity assay that was not affected by direct oral anticoagulants [7]. hFVa (2 μM, Prolytix) or FVm-RRQ (0.2 μM; RRQ denotes Arg^306^, Arg^506^, and Gln^679^) was incubated with human APC (0.1 μM) and phospholipid vesicles (1,2-dioleoyl-sn-glycero-3-phosphoserine/1,2-dioleoyl-sn-glycero-3-phosphocholine/1,2-dioleoyl-sn-glycero-3-phosphoethanolamine, 10:70:20; 25 μM) prepared in HNBSACa^2+^ buffer (25 mM 4-[2-hydroxyethyl]-1-piperazineethanesulfonic acid, 150 mM NaCl, 5 mM CaCl_2_, 0.5% bovine serum albumin, pH 7.5) at 37 °C for 10 minutes in a total reaction volume of 50 μL. The reaction was stopped by adding EDTA buffer (10 mM). Phospholipid vesicles were prepared using an Avanti Mini-Extruder, as previously described [8]. First, we tested the immunoreactivity of these mAbs by Western blot analysis. Reacted samples (FVa, 0.16 pmol/lane; FVm, 0.8 pmol/lane) were subjected to sodium dodecyl sulfate–polyacrylamide gel electrophoresis (5%-20% gradient gel, Wako Pure Chemical) under reducing conditions and electrotransferred to membranes. After blocking with 5% skim milk or 2% bovine serum albumin in phosphate-buffered saline-Tween 20, membranes were incubated with an anti-FV mAb (#AHV-5146, Prolytix) followed by horseradish peroxidase (HRP)-labeled anti-mouse IgG (Kirkegaard & Perry Laboratories), or 0.25 μg/mL HRP-labeled 58CC07 or 58CC09. APC cleavage of FVm-RRQ and hFVa at Arg^306^ yielded an approximately 28 kDa fragment. The anti-FV mAb reacted with the approximately 175 kDa (intact FVm) and 28 kDa (APC-cleaved fragment) bands (Figure 1A). In contrast, 58CC07 and 58CC09 reacted only with the approximately 28 kDa bands derived from FVm and hFVa (Figure 1A, B). Next, we performed a sandwich ELISA using the anti-FV mAb and HRP-labeled 58CC07 or 58CC09. A 96-well plate was coated with 5 μg/mL anti-FV mAb in 50 mM carbonate-bicarbonate buffer (pH 9.6) overnight at 4 °C. After washing with phosphate-buffered saline-Tween 20, the plate was incubated with 25-fold diluted FVm samples, as described above, for 1 hour at room temperature. After washing, the plate was incubated with 0.5 μg/mL HRP-labeled 58CC07 or 58CC09 for 1 hour. After washing, 3,3′,5,5′-tetramethylbenzidine substrate solution was added. The reaction was stopped with 1 N HCl, and absorbance was measured at 450 nm using a SpectraMax i3X plate reader (Molecular Devices). Absorbances of APC-cleaved FVm (mean ± SD, 58CC07: 1.709 ± 0.034; 58CC09: 1.254 ± 0.031) were significantly higher than those of intact FVm (mean ± SD, 58CC07: 0.018 ± 0.001; 58CC09: 0.000 ± 0.005; Figure 1C). These results indicate that both 58CC07 and 58CC09 specifically recognize the FV Asn^307^ neoepitope generated by enzymatic cleavage of FVm/FVa.

**Figure 1:**
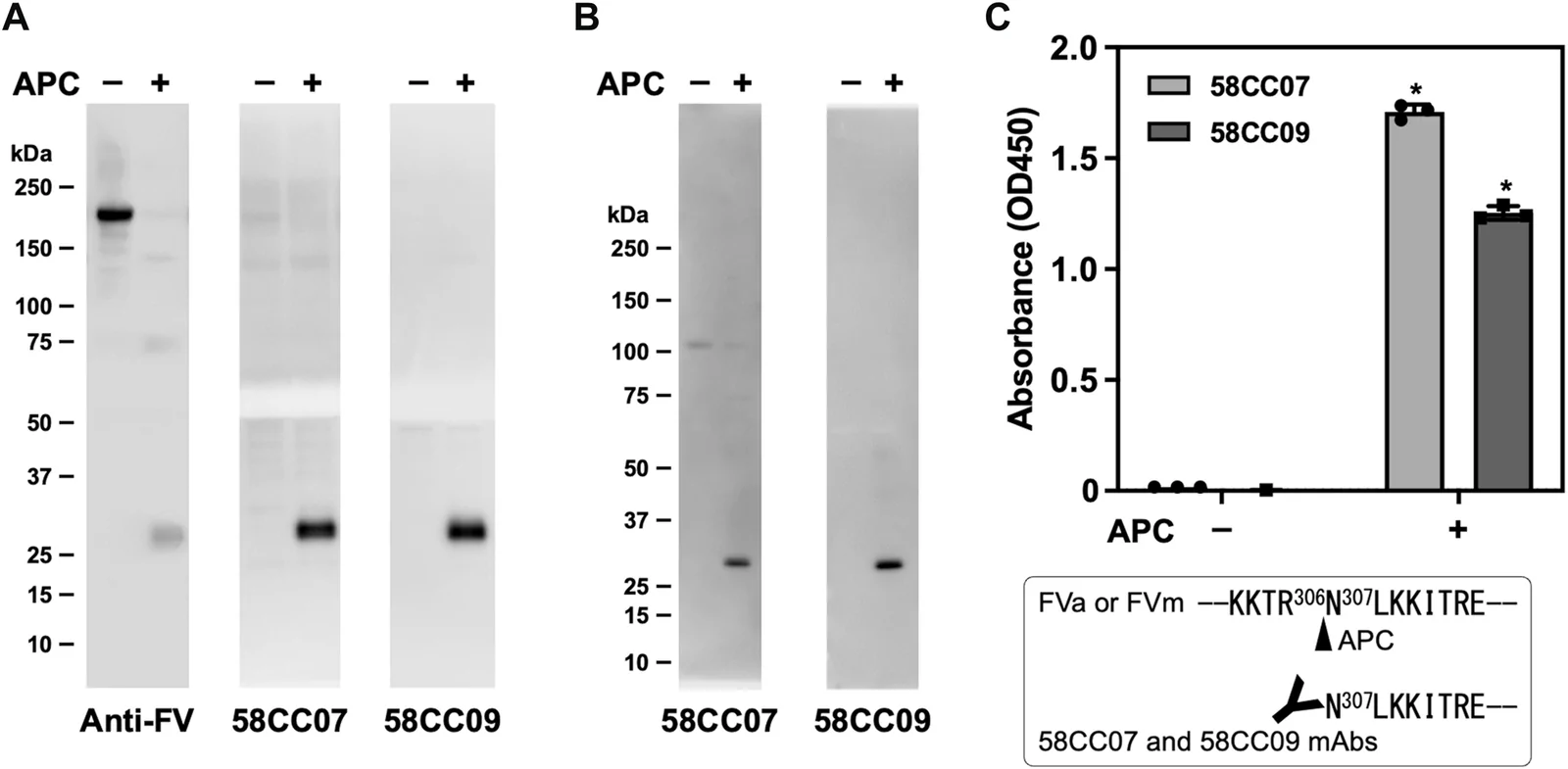
Immunoreactivity of 58CC07 and 58CC09 monoclonal antibodies (mAbs) against factor (F)V fragments generated by activated protein C (APC) cleavage. Western blot analysis of APC-cleaved fragments of (A) purified modified FV (FVm) and (B) human FVa under reducing conditions. Molecular weights are shown on the left. (C) Sandwich enzyme-linked immunosorbent assay using an anti-FV mAb and horseradish peroxidase-labeled FV Asn^307^-specific antibodies. Bars show the mean ± SD (*n* = 3). Statistical analysis was performed using unpaired 2-tailed *t*-tests. The asterisks (∗) indicate significant differences (*P* < .001) between intact and APC-cleaved FVm for each FV Asn^307^-specific mAb. The inset illustrates the Asn^307^ neoepitope generated by APC cleavage at Arg^306^ recognized by the 58CC07 and 58CC09 antibodies. kDa, kilodalton; OD, optical density.

Finally, we confirmed that the mAbs can detect proteolytic cleavage at Arg^306^ of FVa in plasma. We compared BIOPHEN Normal Control Plasma (#223201, Hyphen Biomed; hereafter, normal plasma) with BIOPHEN V-L Control Plasma (#223405, Hyphen Biomed; hereafter, FV-L plasma). The FV-L plasma contains FV Leiden and commonly used in laboratory coagulation assays to assess APC resistance. Plasma samples (5 μL) were incubated with RVV-FV Activator (RVV-V; 10 U/mL, Pentapharm), human APC (0.9 μM), and phospholipid vesicles (25 μM) at 37 °C for 5 minutes in a total reaction volume of 50 μL. No separate chemical stopping reagent was used; after 5 minutes, the samples were immediately diluted 5-fold and subjected to sandwich ELISA using 10 μg/mL 58CC07 or 58CC09 for capture and an anti-FV mAb for detection. When both RVV-V and APC were absent, absorbance in normal and FV-L plasma was low (Figure 2). In the presence of APC, absorbance in normal plasma was significantly higher than in FV-L plasma (mean ± SD, 58CC07: 0.195 ± 0.005 vs 0.139 ± 0.006; 58CC09: 0.318 ± 0.028 vs 0.186 ± 0.016). In the presence of both RVV-V and APC, normal plasma also showed higher absorbance than FV-L plasma (mean ± SD, 58CC07: 0.410 ± 0.015 vs 0.282 ± 0.005; 58CC09: 0.668 ± 0.015 vs 0.451 ± 0.018). These results indicate that FV Asn^307^-specific mAbs can detect APC-mediated cleavage at Arg^306^ in plasma FVa. In this proof-of-principle experiment, the ELISA signal reflecting APC-mediated cleavage was lower in FV-L plasma than in normal plasma under the same assay conditions. These experiments were designed to demonstrate cleavage-specific detection in a plasma matrix, not to establish a diagnostic or quantitative assay. Quantitative measurement would require APC-cleaved FVa or FVm standards of known concentration, determination of assay linearity and sensitivity, normalization for interindividual variation in plasma FV levels, and validation in clinical samples.

**Figure 2:**
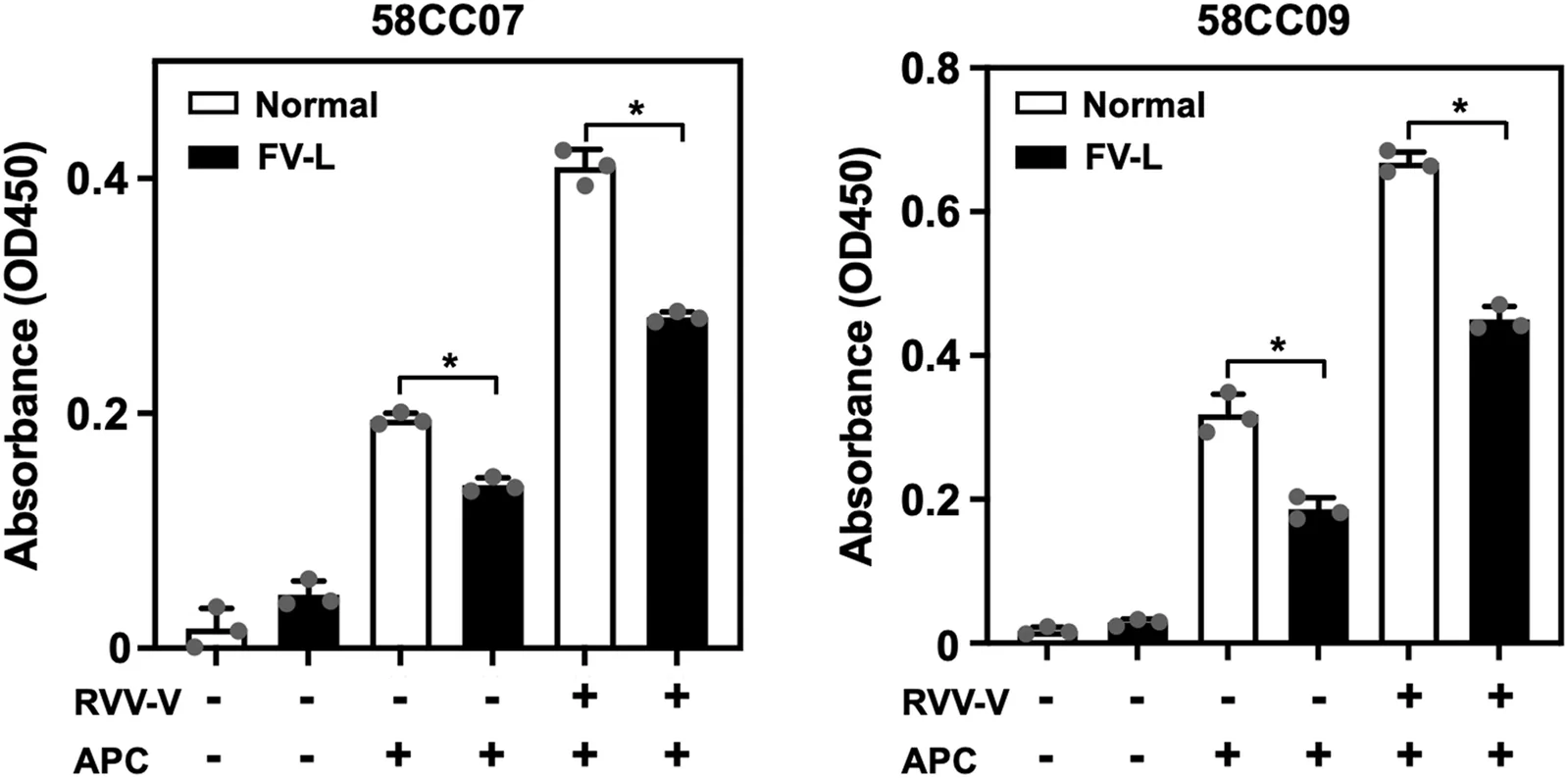
Detection of proteolytic cleavage at Arg^306^ in normal (Normal) plasma and factor (F)V Leiden (FV-L) control plasma. Sandwich enzyme-linked immunosorbent assay using FV Asn^307^-specific antibodies 58CC07 or 58CC09 and an anti-FV monoclonal antibody. Bars show the mean ± SD (*n* = 3). Statistical analysis was performed using unpaired 2-tailed *t*-tests. The asterisks (∗) indicate significant differences (*P* < .01) between Normal and FV-L plasma under the same treatment conditions. APC, activated protein C; OD, optical density; RVV-V, RVV-FV Activator.

Taken together, these results demonstrate that 58CC07 and 58CC09 directly detect the Asn^307^ neoepitope produced by APC-mediated cleavage of FVa at Arg^306^. These mAbs may therefore serve as cleavage-site-specific research reagents for biochemical studies of FV/FVa proteolysis.
